# Diagnostic value of IL-6 for patients with asthma: a meta-analysis

**DOI:** 10.1186/s13223-023-00794-3

**Published:** 2023-05-12

**Authors:** Ruilin Pan, Shougang Kuai, Qingqing Li, Xuming Zhu, Tingting Wang, Yubao Cui

**Affiliations:** 1 Clinical Research Center, The Affiliated Wuxi People’s Hospital of Nanjing Medical University, Wuxi, 214023 Jiangsu Province China; 2 Department of Clinical Laboratory, Huishan District Hospital, WuXi, 214187 Jiangsu Province China; 3 Department of Clinical Laboratory, the Affiliated Wuxi People’s Hospital of Nanjing Medical University, Wuxi, 214023 Jiangsu Province China

**Keywords:** IL-6, Biomarker, Asthma, Meta-analysis

## Abstract

**Background:**

IL-6 is a pleotropic cytokine that acts as a pro-inflammatory mediator and acute-phase response inducer, but has also been reported to possess anti-inflammatory properties. The objective of this study was to assess the validity of serum IL-6 test for diagnosis of asthma.

**Methods:**

A literature search was conducted using PubMed, Embase, and Cochrane library from January 2007 to March 2021 to identify relevant studies. Eleven studies were included in this analysis, involving 1977 patients with asthma and 1591 healthy non-asthmatic controls. The meta-analysis was performed using Review Manager 5.3 software and Stata 16.0. Random effect model or fixed effect model (FEM) was used to estimate the standardized mean differences (SMDs) with 95% confidence intervals (CIs).

**Results:**

The meta-analysis results revealed that the serum IL-6 levels were higher in asthmatic patients than healthy non-asthmatic controls (SMD 1.31, 95% CI 0.82–1.81, P < 0.00001). IL-6 levels are significantly elevated in pediatric patients with asthma (SMD 1.58, 95% CI 0.75–2.41, P = 0.0002) and mildly elevated in adult patients with asthma (SMD 1.08, 95% CI 0.27–1.90, P = 0.009). In addition, a subgroup analysis of asthma disease status showed that IL-6 levels were increased in stable (SMD 0.69, 95% CI 0.28–1.09, P = 0.009) and exacerbation asthma (SMD 2.15, 95% CI 1.79–2.52, P < 0.00001) patients.

**Conclusion:**

The results of this meta-analysis suggest that serum IL-6 levels were significantly elevated in asthmatic patients as compared to normal population. IL-6 levels can be used as an auxiliary indicator to distinguish individuals with asthma from healthy non-asthmatic controls.

**Supplementary Information:**

The online version contains supplementary material available at 10.1186/s13223-023-00794-3.

## Introduction

Asthma is defined as reversible airflow obstruction in the setting of airway inflammation and is characterized by variable symptoms such as wheezing, breathlessness, chest tightness, and cough [1, 2]. Currently, asthma remains an important worldwide health problem in terms of both prevalence and severity in all regions and age group. There are an estimated 235–334 million asthma sufferers worldwide and it is responsible for approximately 250,000 deaths annually [3].

The extent of asthma is usually determined by a severity classification termed to be either intermittent or persistent. Persistent asthma then is classified as mild, moderate, or severe. In addition, asthma is a heterogeneous disease encompassing different phenotypes. asthma patients can be classified into allergic, nonallergic, occupational, aspirin-exacerbated respiratory disease, potentially fatal, exercise-induced, and cough variant asthma [4]. Traditionally asthma diagnosis is predominantly based on the combined of clinical history, presence of typical symptoms, and objective tests of lung function or airway hyperresponsiveness [5]. However, there are some patients who failed to get the lung function and airway hyperresponsiveness objective measurement, which may lead to inappropriate underdiagnosis. Therefore, it is important to find new potential asthma biomarkers, which could contribute to a better understanding of the pathophysiological mechanisms of asthma and to the search for new therapeutic targets.

Specific biomarkers may guide diagnosis, treatment, and predict treatment responses [6]. IL-6 is a typical cytokine with roles in the immune response, inflammation, hematopoiesis and in the endocrine and nervous systems [7]. In early 1980, a study found that there was an active factor that could induce immunoglobulin (Ig) production in Epstein-Barr virus (EBV) -transformed B lymphoblastoid cell lines [8, 9]. Furthermore, the plasmacytoma/hybridoma/myeloma growth factor and the hepatocyte stimulating factor were also found to be identical to this factor [10, 11]. The factor was later named as “interleukin 6” [12]. IL-6 is produced by cells of the innate immune system, B cells, and a small number of CD4 effector Th cells. In addition, IL-6 is also secreted by non-leukocytes such as endothelial cells, fibroblasts, astrocytes, epithelial cells and some malignant cells [13]. These cells can be stimulated to produce IL-6 by actions that promote cell stress or damage (such as UV, irradiation, ROS, microbial products, viruses, or other pro-inflammatory cytokines)[14]. IL-6, which is rapidly and transiently produced in response to infections and tissue injurys, promotes host defense by stimulating acute phase responses, hematopoiesis, and immune responses. Therefore, IL-6 has long been considered a general marker of inflammation. However, IL-6 is now found to be a marker of certain inflammatory diseases. The last decade or so has seen an increasing number of studies shedding light on IL-6 as key cell signaling mode in asthma-related pathways and may play a role as a biomarker of asthma [15]. However, most of these studies have small sample sizes, and a single study may lack sufficient statistical relevance to detect the potentially subtle effects of the IL-6 levels on asthma. Thus, we performed a meta-analysis to accurately investigate the association between IL-6 and asthma.

## Methods

### Search strategy

We searched several commonly used databases (from January 1, 2007 until March 8, 2021) from PubMed, Embase, and the Cochrane Library. A combination of searching terms were used to search those three electronic databases, relating to the following two concepts: (1) IL-6 (‘Interleukin-6 ’ OR ‘ B cell stimulatory factor 2 ’ OR ‘B cell differentiation factor 2’ OR ‘BSF-2’OR‘IL6’OR ‘hybridoma growth factor’ OR ‘plasmacytoma growth factor ’OR ‘hepatocyte stimulating factor’ OR ‘MGI-2’ OR‘ myeloid differentiation inducing protein ’OR ‘interferon beta 2’ ) and (2) asthma (‘Asthma’ OR‘ bronchial asthma’ OR ‘Asthmas’ OR ‘asthma bronchial’). The searches were restricted to studies of only human subjects, and only articles in the English and Chinese language were applied. The references of included studies or reviews were also checked for additional reports.

### Study selection

The inclusion criteria were defined as follows: (1) patients with asthma were studied, (2) express IL-6 levels as mean ± standard deviation, median and range, or median and interquartile range, (3) include patients with asthma and controls, who had no history of asthma or other respiratory disease, (4) if there was duplication of data, only the most complete and recent study was included, and (5) the study design is a case-control study .

### Data extraction and quality assessment

The general characteristics of the study were extracted using a standardized data extraction form: publication information (first author’s name, Publication year), study population, sample size, IL-6 measurement, and IL-6 levels and units(ng/L) in patients with asthma and controls. In terms of quality evaluation, we applied the Newcastle–Ottawa Quality Assessment Scale, with a total score of 0–3, 4–6, and 7–9 considered low, moderate, and high quality, respectively.

### Statistical analysis

The meta-analysis was performed using Review Manager 5.3 software and Stata 16.0. The means and standard deviation were pooled and calculated between patients with asthma and controls. Wood et al. reported median and interquartile range, we analyzed the mean and standard deviation mathematically [16, 17]. In addition, we analyzed the age of patients with asthma, including adults and children with asthma, as well as the status of asthmatic patients (asthma exacerbation and stable asthma). Exacerbation of asthma was defined as the sudden onset of symptoms, such as wheezing, shortness of breath, cough and chest tightness, or a worsening of the original symptoms, often with dyspnea, characterized by a decrease in forced expiratory volume in one second (FEV1). Stable asthma was defined as stable symptoms and lung function maintained for at least 4 weeks. Heterogeneity was assessed using a chi-squared Q test and I-squared statistics. If PQ < 0.1 or I ^2^> 50%, the heterogeneity was considered significant, and a random effects model (REM) was used,otherwise a fixed effect model (FEM) was used. If the mean level differences were significant across studies, or different units were used, standardized mean difference (SMD) was used to estimate the effect size. In the included studies, the serum IL-6 were measured in ELISA with different source reagent, and the differences in the mean levels of IL-6 were considered significant, therefore, SMD in serum IL-6 was used to estimate the effect size. Publication bias was assessed by examining the funnel plot. A sensitivity analysis was performed to explore the stability of the meta-analysis.

## Results

### The general data

**Fig. 1:**
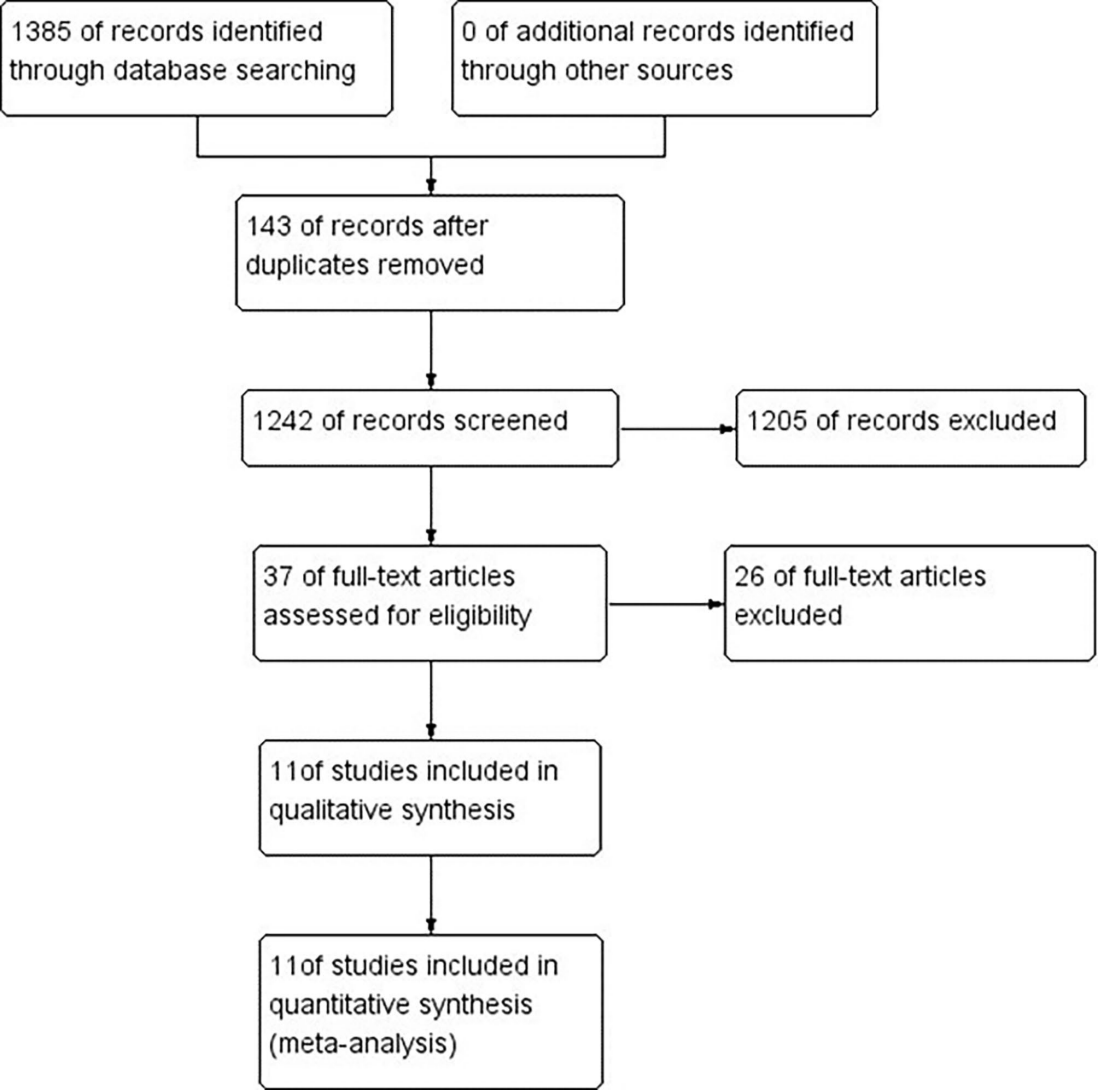
Flow chart of searching the relevant studies used in this meta-analysis

The main characteristics of the included studies are summarized in Table 1 and the steps for screening and the study selection procedure are presented in Fig. 1 .We initially identified 1385 records in the electronic databases and after removing duplicates, 1242 records were left for title and abstract screening. Finally, we obtained a total of eleven relevant studies [18–28] on IL-6, which consist of 1977 patients with asthma and 1591 healthy non-asthmatic controls.

**Table 1:** The general data of included documents

Study	Year	Samples	Country	Size(A/C)	Sex(M/F)		Method
					Asthma	Control	
Ding [18]	2015	Serum	China	120/120	62/58	60/60	ELISA
Radulovic [19]	2015	Serum	America	12/10	9/3	10/ NA	ELISA
Canöz [20]	2008	Serum	Turkey	54/42	NA /54	NR	ELISA
Naik [21]	2017	Serum	India	50/30	27/23	14/16	ELISA
Domvri [22]	2019	Serum	Greece	90/30	31/59	10/20	ELISA
Wood [23]	2012	Serum	Australia	132/83	55/77	31/52	ELISA
Ma [24]	2019	Serum	China	192/130	97/95	67/63	ELISA
Zhang [25]	2018	Serum	China	70/25	25/45	8/15	ELISA
Cui [26]	2017	Serum	China	1158/1075	719/439	686/389	ELISA
Fabian [27]	2011	Serum	Hungary	35/21	28/7	16/5	ELISA
Cui [28]	2014	Serum	China	64/25	NR	NR	ELISA

Abbreviations: ELISA, enzyme-linked immunosorbent assay; A, asthma; C, control; M, male; F, female; NR, not report; NA, not applicable

Note that enzyme-linked immunosorbent assay (ELISA) was used in all included studies. According to the Newcastle–Ottawa Scale, eleven studies had high quality. Low-quality studies were not included in this meta-analysis.

### Correlation of IL-6 in patients with asthma

**Fig. 2:**
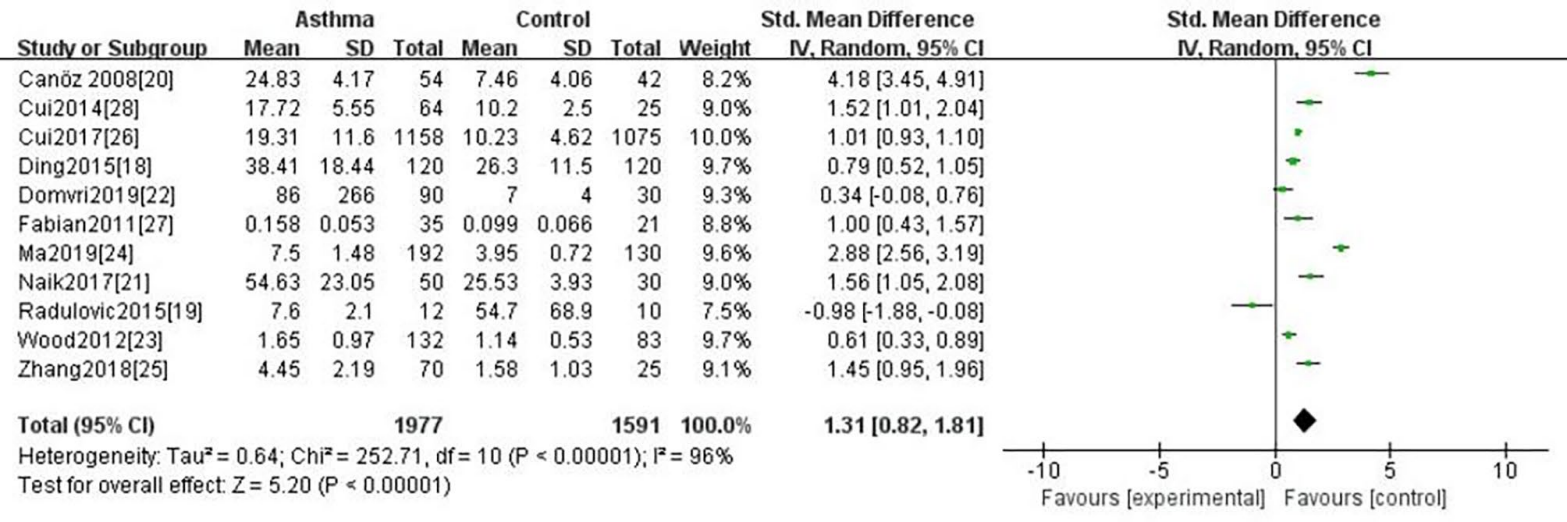
Comparison of serum IL-6 levels between asthma patients and healthy non-asthmatic controls in the included studies

Substantial heterogeneity was observed (I^2^ = 96%) among studies. Thus, our analysis was performed using a random-effects model. Figure 2 provided a comparison of serum IL-6 levels between asthma patients and controls in the included studies. The pooled SMDs revealed that serum IL-6 levels were significantly higher in the asthma group as compared to the healthy non-asthmatic controls group (SMD = 1.31, 95% CI: 0.82–1.81, P < 0.00001).

### Sensitivity analysis and publication bias

The funnel plots were substantially asymmetric **(Supplementary Fig. **1), indicating the presence of a possible publication bias. **Supplementary Fig. **2 showed estimates in the random effects model, with the middle line representing the calculated median (2.01) for all samples, the left and right lines representing the lower (1.40) and higher (3.58) of 95% confidence intervals, respectively, and each circle representing the new mean value obtained after removing a given study from the pool. This indicated that all data were within the 95% confidence intervals for the total data set and that no single study had a large effect on the mean value.

### Subgroup analysis

**Fig. 3:**
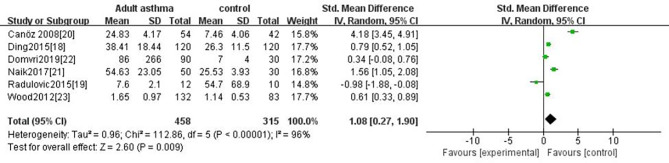
Forest plot of comparison serum IL-6 levels of adult asthma versus the healthy non-asthmatic controls

**Fig. 4:**
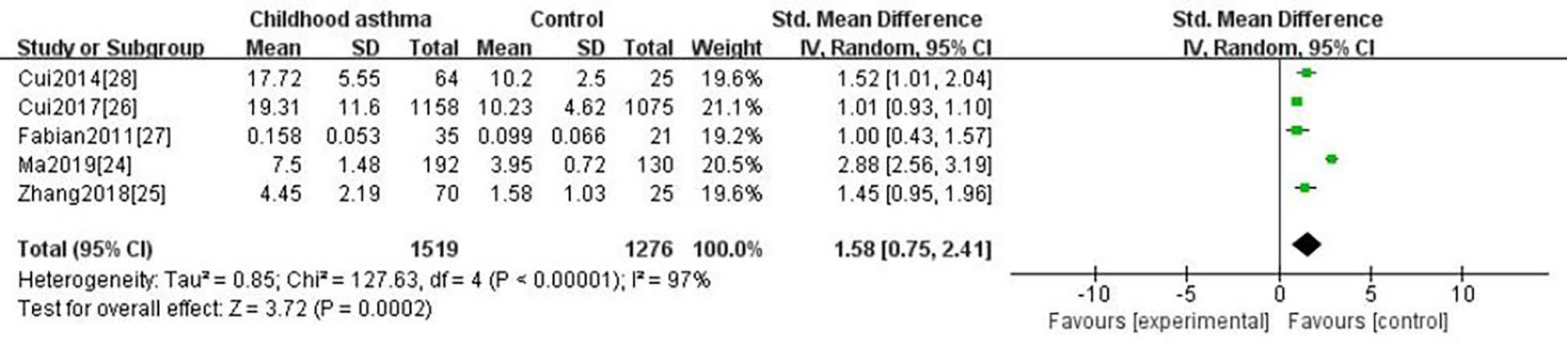
Forest plot of comparison serum IL-6 levels of childhood asthma versus the healthy non-asthmatic controls

Based on the age of patients (Children/Adult), we conducted subgroup analyses to evaluate the association between the age of patients and IL-6 levels. As in a subgroup of adult asthma, six studies [18–23] were included and provided a total number of 458 participants. The estimated SMD (1.08, 95% CI 0.27–1.90, P = 0.009) indicated that serum IL-6 levels were higher in asthmatics as compared to control group (Fig. 3).Five studies [24–28]were included in the subgroup of children with asthma, contributing 1,519 participants for this meta-analysis. The estimated SMD of 1.58 (95% CI 0.75–2.41, P < 0.0002) showed that serum IL-6 levels were higher than those in the control group (Fig. 4).

**Fig. 5:**
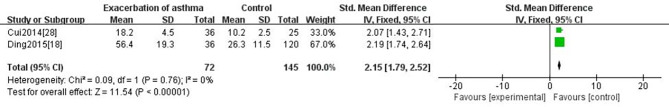
Forest plot of comparison serum levels of IL-6 in the asthma exacerbation group versus the healthy non-asthmatic controls

**Fig. 6:**
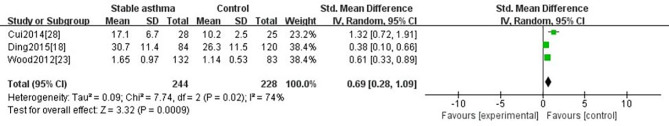
Forest plot of comparison serum levels of IL-6 in the stable asthma group versus the healthy non-asthmatic controls

Considering the asthma status ( asthma exacerbation/ stable asthma), the results of subgroup analysis of asthma exacerbation showed that serum IL-6 levels were significantly higher in patients with asthma exacerbation than in healthy non-asthmatic controls, with a pooled SMD of 2.15 (95% CI 1.79–2.52, P < 0.00001)[18, 28] (Fig. 5). The similar results were also found in stable asthma (SMD 0.69, 95% CI 0.28–1.09, P = 0.0009)[18, 23, 28] (Fig. 6).

### Meta-regression analysis

Due to the high heterogeneity in Fig. 2, we conducted multivariate meta-regression analysis to study the confounding factors that may lead to this phenomenon. The results showed that year, country and age were not the source of the heterogeneity, which may be caused by multiple factors.

## Discussion

The present study was conducted to investigate the potential role of IL-6 in asthma patients. The main findings of this meta-analysis are (1) patients with asthma had higher serum IL-6 concentrations than those in controls; (2) Serum IL-6 levels are elevated in both adult and pediatric asthmatics compared to controls, especially in pediatric asthmatics; (3) Serum IL-6 levels were higher in patients with exacerbation and stable asthma than in healthy non-asthmatic controls.

IL-6 levels usually rise when cells are stressed or injured. The researchers found that serum IL-6 levels were elevated, which was related to inflammatory diseases [29]. It is acknowledged that airway inflammation is a prominent feature of asthma [30]. Interestingly, lung epithelial cells can secrete cytokines such as IL-6 and regulate immunity [31]. It has also been reported that IL-6 is overexpressed in bronchial epithelial cells in adults and children with asthma [32]. In addition, Asthma is treated with inhaled corticosteroids which shut down the expression of asthma-related cytokines such as IL-6[33]. Taken together,these results suggest that serum IL-6 levels may be elevated in asthmatic patients. There is growing evidence that IL-6 may also play an important role in the initial development and subsequent progression of asthma [34, 35].

In present meta-analysis, we observed significant heterogeneity in these studies. This heterogeneity remained after the subgroup analysis, suggesting that the study population, measure reagents, age and other covariates may be responsible for it. Therefore, we performed a meta-regression analysis to explore possible sources of heterogeneity, and the results showed that none of these factors was a source of heterogeneity. The ELISA kits in the study were purchased from different manufacturers. Thus, among the included studies, the sensitivity of the measurement reagents was different, which can be a possible factor for heterogeneity. Furthermore, Sensitivity analyses indicated that the pooled SMD were not excessively affected by a single study.

This paper has several limitations. First of all, only articles in the English and Chinese language studies are picked in this meta-analysis, and articles in other languages that meet the inclusion criteria may be missed, which may lead to publication bias. Secondly, the sample size of the eleven articles included in this study is small, which may be one of the reasons to explain the existence of publication bias.

## Conclusion

In summary, although limited studies have reported serum IL-6 level in asthma, our findings associate higher levels of IL-6 in patients with asthma, especially in children with asthma. Thus, IL-6 levels can be used as an auxiliary indicator to distinguish individuals with asthma from healthy non-asthmatic controls.

## Electronic supplementary material

Below is the link to the electronic supplementary material.

**Supplementary Figure 1**. Funnel plot with pseudo 95% confidence intervals

**Supplementary Figure 2**. Meta-analysis random-effects estimates for all the included studies

## Data Availability

The datasets used and analyzed during the current study can available from the corresponding author on reasonable request.
