# Supplementary figures and images for: Diagnostic value of IL-6 for patients with asthma: a meta-analysis
Source: Allergy Asthma Clin Immunol. 2023 May 12;19:39. doi: 10.1186/s13223-023-00794-3 (PMC10182700; doi:10.1186/s13223-023-00794-3)

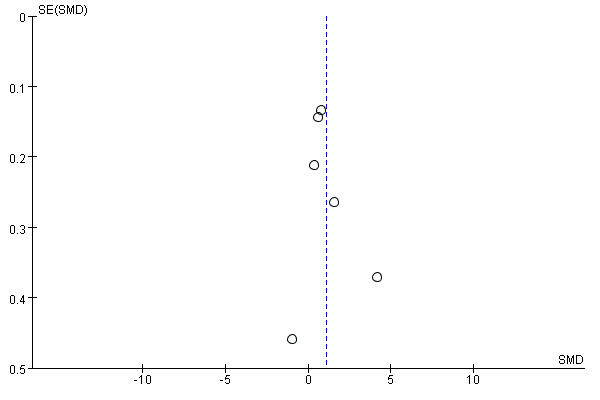

Supplement: Supplementary file 1 — Supplementary Figure 1. Funnel plot with pseudo 95% confidence intervals [file 13223_2023_794_MOESM1_ESM.jpg]

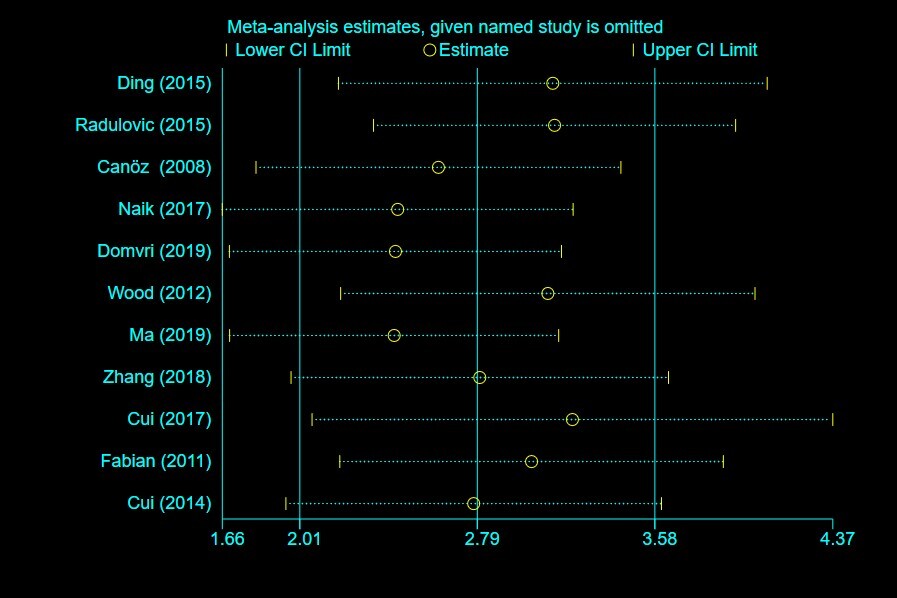

Supplement: Supplementary file 2 — Supplementary Figure 2. Meta-analysis random-effects estimates for all the included studies [file 13223_2023_794_MOESM2_ESM.jpg]
